# Differential association between obesity and coronary artery disease according to the presence of diabetes in a Korean population

**DOI:** 10.1186/1758-5996-6-134

**Published:** 2014-12-05

**Authors:** Ki-Bum Won, Hyuk-Jae Chang, Hiroyuki Niinuma, Jimin Sung, In-Jeong Cho, Chi-Young Shim, Geu-Ru Hong, Young Jin Kim, Byung-Wook Choi, Namsik Chung

**Affiliations:** Division of Cardiology, Yonsei Cardiovascular Center, Yonsei University College of Medicine, 50 Yonsei-ro, Seodaemun-gu, Seoul, 120-752 Republic of Korea; Division of Cardiology, St. Luke’s International Hospital, Tokyo, Japan; Graduate School of Health and Welfare CHA University, Seongnam, Republic of Korea; Division of Radiology, Yonsei Cardiovascular Center, Yonsei University College of Medicine, Seoul, Republic of Korea; Severance Biomedical Science Institute, Seoul, Republic of Korea

**Keywords:** Obesity, Diabetes, Coronary artery disease, Cardiac computed tomographic angiography

## Abstract

**Background:**

Coronary artery disease (CAD) is a major cardiovascular complication in diabetic patients. Despite the significant association between obesity and diabetes, the majority of the diabetic subjects are not obese in an Asian population. This study evaluated the association between obesity and coronary artery disease (CAD) according to the diabetes status in a Korean population.

**Methods:**

The association between obesity and CAD using the parameters of any plaque, obstructive plaque, and coronary artery calcium score (CACS) >100 according to the presence of diabetes was evaluated in 7,234 Korean adults who underwent multi-detector computed tomography for general health evaluations. Obesity was defined as a body mass index (BMI) ≥25 kg/m^2^.

**Results:**

The prevalence of obesity was significantly higher in diabetic subjects than in non-diabetic subjects, but the majority of the diabetic subjects were non-obese (48% vs. 37%, p <0.001). The incidence of any plaque (58% vs. 29%), obstructive plaque (20% vs. 6%), and CACS >100 (20% vs. 6%) were significantly higher in diabetic patients than in non-diabetic subjects (p <0.001, respectively). Incidence of any plaque (33% vs. 26%, p <0.001), obstructive plaque (7% vs. 6%, p = 0.014), and CACS >100 (8% vs. 6%, p = 0.002) was significantly higher in non-diabetic subjects with obesity than in those without obesity, but the incidence of all coronary parameters was not different in diabetic subjects according to the obesity status. After adjusting for confounding risk factors including age, gender, hypertension, dyslipidemia, current smoking, and mild renal dysfunction, obesity was independently associated with increased risks of any plaque (OR 1.14) and CACS >100 (OR 1.31) only in non-diabetic subjects (p <0.05, respectively). Multiple logistic regression models revealed that diabetes was independently associated with all coronary parameters.

**Conclusion:**

Despite a significantly higher prevalence of obesity in diabetic subjects than in non-diabetic subjects, obesity is associated with the presence of any plaque and severe coronary calcification only in subjects without established diabetes among Korean population.

## Background

Diabetes is significantly associated with an increased risk of coronary artery disease (CAD). Although the pathogenesis of diabetes is complicated by multiple metabolism-related problems, a deterioration of insulin secretion and an aggravation of insulin resistance are 2 central defects in the pathogenesis of diabetes [1, 2]. It is obvious that obesity is one of the major factors for insulin resistance. However, the criterion of obesity is dependent on ethnicity, and the prevalence of obesity differs according to ethnicity. In addition, despite the substantial increases in the prevalence of obesity and diabetes in Asia, the clinical features of the development of diabetes in Asia are explicitly different from those in other parts of the world, with diabetes developing in a much shorter time, at a younger age, and in subjects with much lower body mass index (BMI) in Asia [3]. The majority of individuals with diabetes are not obese, even with obesity defined as a BMI of more than 25 kg/m^2^, and significant weight loss is observed during the course of the development of diabetes in Korean population [4]. Furthermore, several studies on the pathogenesis of type 2 diabetes reported that impaired insulin secretion is more prominent than insulin resistance, even in the status of impaired glucose tolerance [5, 6]. Accordingly, whether obesity is an independent predictor for CAD in Asian diabetic subjects may be an important issue, but data are scarce in Asian populations.

The coronary artery calcium (CAC) score (CACS), which has developed to quantify the extent of CAC [7], is a good marker of coronary atherosclerosis [8, 9]. CACS is closely correlated with the volume of coronary plaque measured by autopsy and is considered a surrogate marker for the overall coronary plaque burden [10–12]. A few previous studies investigated the relationship between obesity and CAC, but the results were inconsistent. Some reported a positive and independent association [13–15], while others reported a null [16–18], or even an inverse association [19]. Furthermore, most relevant studies were conducted in a Western population, in which obesity and CAD are more prevalent compared with other populations such as East Asian. In addition, they evaluated the association between obesity and CAC without considering the status of diabetes. Recently, coronary computed tomographic angiography (CCTA) was introduced as a novel noninvasive imaging approach for evaluating coronary atherosclerosis [20, 21], and it has high diagnostic accuracy in detecting CAD [22, 23]. Therefore, we investigated the association between obesity and coronary atherosclerosis according to diabetes status using the noninvasive CCTA in Korean subjects with near-normal kidney function.

## Methods

### Subjects

This cross-sectional study consisted of 8,648 consecutive subjects who had undergone CCTA evaluation with 64-slice multi-detector computed tomography (MDCT) from January 2004 to April 2009 at Severance Cardiovascular Hospital. All subjects were referred for general health evaluations with following various indications: symptoms such as chest discomfort, dyspnea, or fatigue; no symptoms but having abnormal electrocardiographic test, previous history of peripheral artery disease or cerebrovascular disease, or the presence of multiple cardiovascular risk factors. Subjects were excluded for any one of the following criteria: (a) age < 30 years (n = 69); (b) established chronic kidney disease or glomerular filtration rate (GFR) <60 ml/min/1.73 m^2^, estimated by the modification of diet in renal disease formula (n = 959); and (c) insufficient medical records (n = 386). As a result, 7,234 subjects were included in this study. The study protocol was approved by the local ethics committee of our institution.

### Protocol of MDCT

Data acquisition and image post-processing were performed in accordance with the Society of Cardiovascular Computed Tomography guidelines on CCTA acquisition [24, 25]. Briefly, subjects with an initial heart rate ≥65 beats/min before MDCT received a single oral dose of 50 mg of metoprolol (Betaloc; Yuhan, Seoul, Korea) 1–2 h before CT examination unless β-adrenergic blocking agents were contraindicated (overt heart failure, atrioventricular conduction abnormalities, or bronchial asthma). Subjects were scanned with a 64-slice CT scanner (Sensation 64; Siemens Medical Solutions, Forchheim, Germany). Initially, a non-enhanced prospective electrocardiogram (ECG)-gated scan to evaluate CACS was performed with the following parameters: rotation time of 330 ms, slice collimation of 0.6 mm, slice width of 3.0 mm, tube voltage of 100–120 kV, tube current of 50 mA, and table feed/scan of 18 mm. CCTA was then performed using retrospective ECG-gating with the following scan parameters: rotation time of 330 ms, slice collimation of 64 × 0.6 mm, tube voltage of 100–120 kV, tube current of 400–800 mA depending on patient size, table feed/scan of 3.8 mm, and pitch factor of 0.2. ECG-based tube current modulation was applied to 65% of the R–R interval. A real-time bolus-tracking technique was applied to trigger scan initiation. The total estimated average radiation dose for the multi-slice CT protocol was 8.7 ± 1.5 mSv. Contrast enhancement was achieved using 60 mL of iopamidol (370 mg iodine/mL, Iopamiro; Bracco, Milan, Italy) injected at 5 mL/s, followed by an injection of 30 mL of diluted contrast (the ratio of saline to contrast agent was 7:3) and then 30 mL of saline at 5 mL/s with a power injector (Envision CT; Medrad, Indianola, PA) via an antecubital vein.

Image reconstruction was carried out on the scanner workstation using commercially available software (Wizard; Siemens Medical Solutions, Forchheim, Germany). Axial images were reconstructed retrospectively at 65% of the RR interval for each cardiac cycle. If artifacts were present, then additional data sets were obtained for various points of the cardiac cycle, and the data set with the smallest artifact was selected for further analysis. The reconstructed image data sets were transferred to an off-line workstation (Aquarius Workstation; TeraRecon, Inc., San Mateo, CA). Each lesion identified was examined using maximum-intensity projection and multiplanar reconstruction techniques on a short axis and along multiple longitudinal axes. Lesions were classified by the maximal stenosis of the luminal diameter observed on any plane.

### Measurement of CT variables

CCTA data were evaluated by 2 experienced cardiac radiologists (Y.J.K. and B.W.C., who have 6 and 9 years of experience in cardiac CT, respectively). This study primarily evaluated the presence of any plaque, obstructive plaque, and CACS >100. Both any plaque and obstructive plaque were divided into 2 subtypes according to the presence of coronary calcification following calcified or mixed plaque and non-calcified plaque, respectively. CACS was measured using a previously described method [7]. Because the frequency of CACS >100 in the Asian population is known to be lower than that in Caucasians, African-Americans, and Hispanics [26], we used CACS >100 as the parameter for identifying severe coronary calcification.

Plaque was defined as structures >1 mm^2^ within and/or adjacent to the vessel lumen that were clearly distinguished from the lumen and surrounding pericardial tissue. Obstructive plaque was defined as plaque with ≥50% luminal diameter stenosis. Calcified plaque was defined as plaque occupied by calcified tissue for ≥50% of the plaque area (density >130 Hounsfield units in native scans). Mixed calcified plaque was defined as plaque in which <50% of the area is occupied by calcified tissue. Plaque without any calcium was defined as non-calcified plaque. CAD was defined as the presence of any coronary plaque and calcium identified by CCTA.

### Measurement of clinical variables

Medical histories of hypertension, dyslipidemia, diabetes, and smoking status were systematically acquired for the subjects. Height, weight, and blood pressure were measured during visits. All blood samples, including those for triglycerides, high-density lipoprotein (HDL) cholesterol, low-density lipoprotein (LDL) cholesterol, and glucose, were obtained after a 12-h fast on the day of the CT scan as part of the clinical work-up. BMI was calculated as weight (kg) ÷ height (m^2^), and obesity was defined as a BMI ≥25 kg/m^2^. Hypertension was defined as systolic blood pressure ≥140 mmHg and/or diastolic blood pressure ≥90 mmHg or the use of antihypertensive treatment. Dyslipidemia was defined as total cholesterol ≥240 mg/dL, LDL ≥130 mg/dL, HDL ≤40 mg/dL, TG ≥150 mg/dL, or treatment with lipid-lowering agents. Current smoking history was considered present if subjects consistently smoked or smoked within 1 month before the study. Mild renal dysfunction was defined as a GFR of 60–89 ml/min/1.73 m^2^. Diabetes was defined as fasting glucose ≥126 mg/dL, the receipt of antidiabetic treatment, or a referral diagnosis of diabetes.

### Statistical analysis

Continuous variables are expressed as the mean ± SD or medians and interquartile range according to the distribution. Categorical variables are presented as n (%). Continuous variables were compared using an independent t-test or Mann-Whitney’s U-test, and categorical variables were compared using the χ^2^ test or Fisher’s exact test, as appropriate. Univariate and multivariate logistic regression analysis for evaluating the impact of obesity and BMI on the presence of any plaque, obstructive plaque, and CACS <100 were performed separately for subjects with and without diabetes. Multivariate logistic regression analysis was adjusted for confounding risk factors including age, gender, hypertension, dyslipidemia, current smoking, and mild renal dysfunction. And, multivariate logistic models were analyzed to identify the impact of diabetes on the presence of any plaque, obstructive plaque, and CACS <100. The covariate-adjusted odds ratio (OR) and 95% confidence intervals (CI) for each parameter were calculated. SPSS version 18 (SPSS Inc., Chicago, IL) was used for all statistical analyses, and p <0.05 was considered significant.

## Results

**Figure 1:**
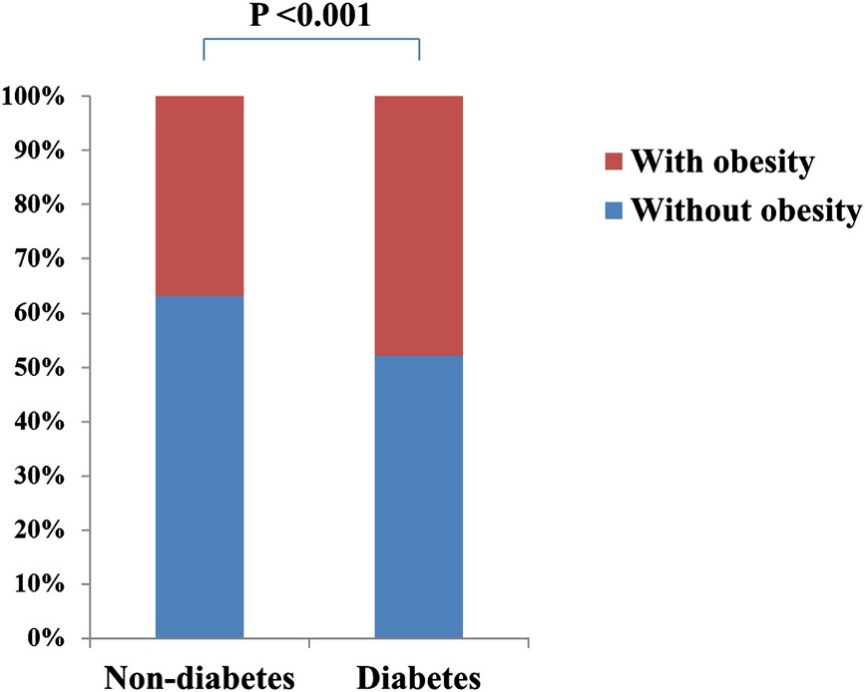
**Comparison of the prevalence of obesity according to the diabetes status.**

**Figure 2:**
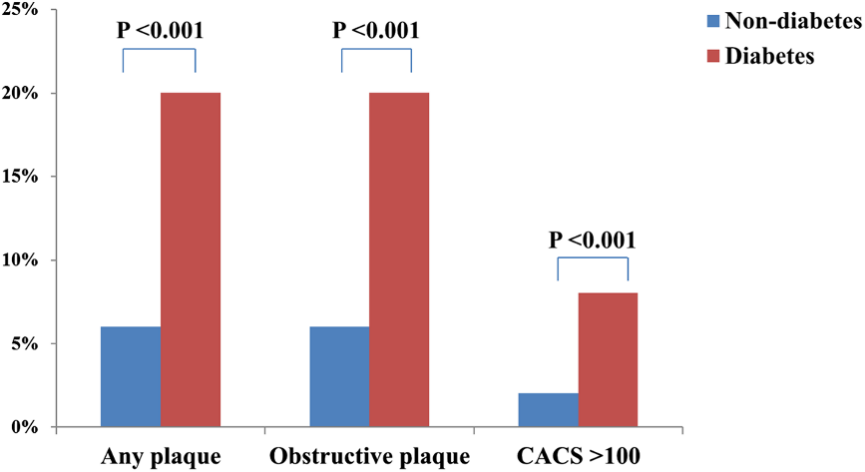
**Comparison of the incidence of coronary parameters according to the diabetes status.**

The clinical characteristics of the 7,234 subjects (52 ± 10 years; 57% men) are listed in Table 1. There were 6,345 non-diabetic (88%) and 889 diabetic subjects (12%). The overall prevalence of obesity in the present study was 38%. The prevalence of obesity was significantly higher in diabetic subjects than in non-diabetic subjects (48% vs. 37%, p <0.001), but the majority of diabetic subjects were non-obese (Figure 1). The prevalence of hypertension, and dyslipidemia were significantly higher in diabetic subjects than in non-diabetic subjects (p <0.001, respectively). The incidence of any plaque (58% vs. 29%), obstructive plaque (20% vs. 6%), and CACS >100 (20% vs. 6%) were significantly higher in diabetic patients than in non-diabetic subjects (p <0.001, respectively) (Figure 2).

**Table 1:** **Baseline characteristics**

	Non-diabetes (n = 6,345)	Diabetes (n = 889)	P
Age (years)	52 ± 10	58 ± 9	<0.001
Men, n (%)	3569 (56)	565 (64)	<0.001
BMI (kg/m^2^)	24.1 ± 2.9	25.1 ± 3.1	<0.001
BMI <18.5	99 (2)	10 (1)	
BMI 18.5 – 24.9	3920 (61)	455 (51)	
BMI 25.0 – 29.9	2137 (34)	373 (42)	
BMI ≥30.0	189 (3)	51 (6)	
Obesity, n (%)	2326 (37)	424 (48)	<0.001
Hypertension, n (%)	1716 (27)	427 (49)	<0.001
Current smoking, n (%)	1351 (22)	186 (21)	0.801
Dyslipidemia, n (%)	3253 (52)	560 (64)	<0.001
Total cholesterol (mg/dL)	187 (164, 210)	177 (155, 203)	<0.001
Triglyceride (mg/dL)	121 (87, 170)	135 (97, 187)	<0.001
HDL cholesterol (mg/dL)	50 (43, 58)	48 (41, 54)	<0.001
LDL cholesterol (mg/dL)	119 (97, 141)	111 (89, 137)	0.007
Creatinine (mg/dL)	0.90 (0.80, 1.00)	0.90 (0.80, 1.00)	<0.001
GFR (ml/min/1.73 m^2^)	80 (72, 91)	80 (72, 90)	<0.001
FBS (mg/dL)	94 (88, 101)	127 (107, 149)	<0.001
Any plaque, n (%)	1814 (29)	511 (58)	<0.001
Obstructive plaque, n(%)	401 (6)	174 (20)	<0.001
CACS >100, n (%)	403 (6)	176 (20)	<0.001

Data are expressed as n (%) or mean ± SD. BMI, body mass index; CACS, coronary artery calcium score; FBS, fasting blood sugar; GFR, glomerular filtration rate; HDL, high-density lipoprotein; LDL, low-density lipoprotein.

The incidence of any plaque (33% vs. 26%, p <0.001), obstructive plaque (7% vs. 6%, p = 0.014), and CACS >100 (8% vs. 6%, p = 0.002) was significantly higher in non-diabetic subjects with obesity than those without obesity. However, there were no significant differences in all these parameters according to the obesity in diabetic subjects (Table 2).

**Table 2:** **Comparison of coronary atherosclerosis according to the obesity status in non-diabetic and diabetic subjects**

	Non-diabetes (n = 6,345)		P	Diabetes (n = 889)		P
	Non-obesity (n = 4,019)	Obesity (n = 2,326)		Non-obesity (n = 465)	Obesity (n = 424)	
Any plaque, n (%)	1055 (26)	759 (33)	<0.001	260 (56)	251 (59)	0.323
CMP	785 (19)	586 (26)	<0.001	226 (49)	209 (50)	0.837
NCP	270 (7)	173 (7)	0.278	34 (7)	42 (9)	0.167
Obstructive plaque, n (%)	231 (6)	170 (7)	0.014	87 (19)	87 (21)	0.496
Obstructive CMP	190 (5)	143 (6)	0.015	77 (17)	71 (17)	0.940
Obstructive NCP	41 (1)	27 (1)	0.601	10 (2)	16 (4)	0.151
CACS >100, n (%)	226 (6)	177 (8)	0.002	95 (21)	81 (19)	0.610

Data are expressed as n (%). CACS, coronary artery calcium score; CMP, calcified or mixed plaque; NCP, non-calcified plaque.

**Figure 3:**
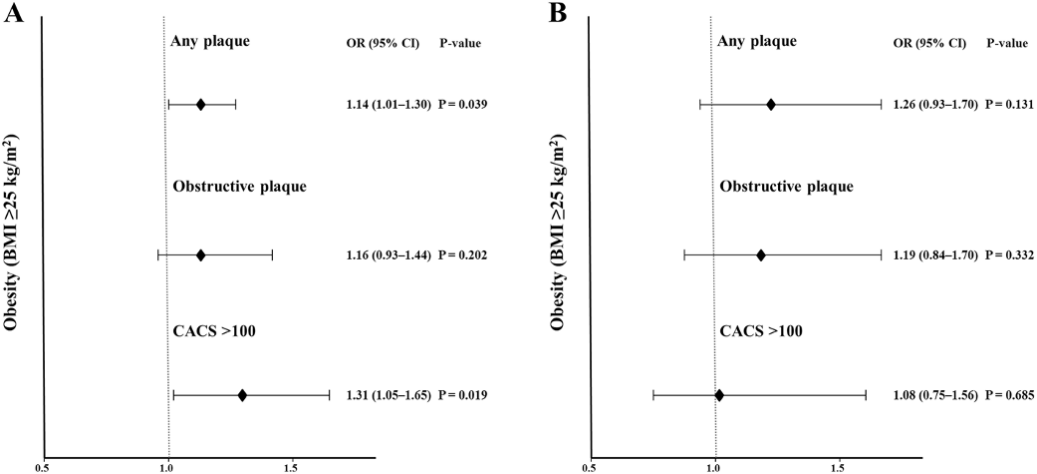
**Association between obesity and coronary parameters. (A)** Non-diabetes, **(B)** diabetes.

Univariate logistic regression analysis revealed that obesity and BMI were significantly associated with increased risks of any plaque (obesity: odd ratio [OR] 1.36, p <0.001; BMI: OR 1.08, p <0.001), obstructive plaque (obesity: OR 1.29, p = 0.014, BMI: OR 1.07, p <0.001), and CACS >100 (obesity: OR 1.38, p = 0.002; BMI: OR 1.07, p <0.001) in non-diabetic subjects. However, neither obesity nor BMI were significantly associated with increased risks of any plaque (obesity: OR 1.14, p = 0.323; BMI: OR 0.98, p = 0.249), obstructive plaque (obesity: OR 1.12, p = 0.496, BMI: OR 0.97, p = 0.342), and CACS >100 (obesity: OR 0.92, p = 0.610; BMI: OR 0.95, p = 0.083) in diabetic subjects. After adjusting for age, gender, hypertension, dyslipidemia, current smoking, and mild renal dysfunction, both obesity and BMI were significantly associated with increased risks of any plaque (obesity: OR 1.14, p = 0.039; BMI: OR 1.04, p = 0.003) and CACS >100 (obesity: OR 1.31, p = 0.019; BMI: OR 1.06, p = 0.004), but not significantly associated with obstructive plaque (obesity: OR 1.16, p = 0.202; BMI: OR 1.04, p = 0.096) in non-diabetic subjects. However, neither obesity nor BMI were significantly associated with increased risks of any plaque (obesity: OR 1.26, p = 0.131; BMI: OR 1.00, p = 0.903), obstructive plaque (obesity: OR 1.19, p = 0.332; BMI: OR 0.99, p = 0.775), and CACS >100 (obesity: OR 1.08, p = 0.685; BMI: OR 0.99, p = 0.685) in diabetic subjects (Table 3 and Figure 3).

**Table 3:** **Impact of obesity and BMI on coronary atherosclerosis according to the diabetes status**

	Any plaque				Obstructive plaque				CACS >100			
	Univariate		Multivariate		Univariate		Multivariate		Univariate		Multivariate	
	OR	95% CI	OR	95% CI	OR	95% CI	OR	95% CI	OR	95% CI	OR	95% CI
Non-diabetes												
Obesity	1.36	1.22–1.52*	1.14	1.01–1.30^‡^	1.29	1.05–1.59^†^	1.16	0.93–1.44	1.38	1.13–1.69^†^	1.31	1.05–1.65^‡^
BMI	1.08	1.06–1.10*	1.04	1.01–1.06^†^	1.07	1.03–1.10*	1.04	0.99–1.08	1.07	1.04–1.11*	1.06	1.02–1.11^†^
Diabetes												
Obesity	1.14	0.88–1.49	1.26	0.93–1.70	1.12	0.81–1.56	1.19	0.84–1.70	0.92	0.66–1.23	1.08	0.75–1.56
BMI	0.98	0.93–1.02	1.00	0.95–1.05	0.97	0.92–1.03	0.99	0.93–1.05	0.95	0.90–1.01	0.99	0.93–1.05

BMI, body mass index; CACS, coronary artery calcium score; CI, confidence interval; OR, odds ratio.

Multivariate analyses were adjusted for age, gender, hypertension, dyslipidemia, current smoking, and mild renal dysfunction.

*P <0.001, ^†^P <0.005, ^‡^P <0.05.

Multivariate logistic models for identifying the impact of diabetes on the presence of any plaque, obstructive plaque, and CACS <100 were analyzed after consecutively adjusting for age, gender, hypertension, BMI, dyslipidemia, current smoking, and mild renal dysfunction. All models illustrated that diabetes had a strong impact on the presence of any plaque, obstructive plaque, and CACS >100 (Table 4).

**Table 4:** **Multiple logistic regression models for identifying the impact of diabetes on coronary atherosclerosis**

	Any plaque		Obstructive plaque		CACS >100	
	OR	95% CI	OR	95% CI	OR	95% CI
Model 1	3.38	2.93–3.90*	3.61	2.97–4.39*	3.65	3.01–4.43*
Model 2	2.37	2.03–2.77*	2.43	1.98–2.98*	2.25	1.82–2.77*
Model 3	2.21	1.88–2.59*	2.32	1.89–2.85*	2.15	1.74–2.67*
Model 4	2.10	1.79–2.47*	2.21	1.80–2.73*	2.08	1.68–2.59*
Model 5	2.05	1.75–2.41*	2.17	1.67–2.76*	2.04	1.64–2.53*
Model 6	2.00	1.70–2.35*	2.12	1.71–2.61*	2.06	1.66–2.56*
Model 7	2.01	1.71–2.36*	2.14	1.73–2.64*	2.08	1.67–2.58*
Model 8	1.96	1.67–2.31*	2.07	1.67–2.56*	2.05	1.65–2.55*

BMI, body mass index; CACS, coronary artery calcium score; CI, confidence interval; OR, odds ratio. *P <0.001.

Model 1: Unadjusted.

Model 2: Adjusted for age.

Model 3: Adjusted for age and gender.

Model 4: Adjusted for age, gender, and hypertension.

Model 5: Adjusted for age, gender, hypertension, and BMI.

Model 6: Adjusted for age, gender, hypertension, BMI, and dyslipidemia.

Model 7: Adjusted for age, gender, hypertension, BMI, dyslipidemia, and current smoking.

Model 8: Adjusted for age, gender, hypertension, BMI, dyslipidemia, current smoking, and mild renal dysfunction.

## Discussion

To the best of our knowledge, the present study is the first to provide information on the differential association between obesity and coronary atherosclerosis according to the presence of diabetes in the Asian population. Diabetes was strongly associated with all coronary parameters including any plaque, obstructive plaque, and CACS >100. Despite a significantly higher prevalence of obesity in diabetic subjects, obesity was independently associated with the presence of CAD and severe coronary calcification only in subjects without established diabetes in a Korean population.

Obesity, a major factor for insulin resistance, is significantly associated with diabetes and CAD [27, 28]. However, the criteria and prevalence of obesity is dependent on ethnicity. Despite the increased prevalence of obesity and diabetes in Asia, the clinical features of the development of diabetes in Asia are different from those in other parts of the world [3]. In Korea, previous studies reported that approximately 65% of subjects with diabetes were non-obese [4], and that impaired insulin secretion was more prominent than insulin resistance in diabetic subjects [5, 6]. Furthermore, a recent study revealed that diabetes had an incremental impact on subclinical atherosclerosis independent of the metabolic syndrome, for which insulin resistance is a major characteristic, in the Korean population [29]. Accordingly, it is important to identify whether obesity is independently associated with CAD in an Asian population with established diabetes.

CAC is a traditional surrogate marker for coronary atherosclerosis because it is significantly related to the coronary plaque burden [10, 11]. It may be that the severity of coronary calcification is somewhat different in an Asian population compared to that in a Western population [26]. However, most studies evaluated the association between obesity and CAC in a Western population with conflicting results [13–19]. CCTA has recently been used for evaluating coronary atherosclerosis because of its high diagnostic accuracy in detecting CAD [22, 23], and only limited studies investigated the relationship of BMI with coronary atherosclerosis using CCTA. Dores et al. [30] reported that BMI was an independent predictor of CAD, but it was not correlated with the severity of CAD in subjects with suspected CAD. Labounty et al. [31] reported that an increased BMI was associated with a greater prevalence, extent, and severity of CAD and was independently associated with an increased risk of myocardial infarction. However, most participants in these studies were from Western populations, and these studies did not evaluate the differential association of BMI with coronary atherosclerosis according to the presence of diabetes. Furthermore, it has not been investigated whether obesity, defined as a BMI ≥25 kg/m^2^, is an independent predictor of CAD in Asian patients with diabetes. We evaluated the association between obesity and coronary atherosclerosis using the parameters of any plaque, obstructive plaque, and CACS >100 according to the presence of diabetes. The prevalence of obesity was significantly higher in diabetic subjects than in non-diabetic subjects, but the majority of diabetic subjects were non-obese in our Korean population. We identified that diabetes was strongly associated with all coronary atherosclerotic parameters. Despite a significantly higher prevalence of obesity in diabetic subjects than in non-diabetic subjects, obesity was independently associated with the presence of CAD and severe coronary calcification in non-diabetic subjects. These results suggest that obesity is not a useful predictor for CAD in subjects with established diabetes, although it was significantly associated with the development of diabetes in a Korean population. These results might imply that the identification of newly developing diabetes might be important in non-diabetic subjects with obesity; however, considering the incremental impact of diabetes on coronary atherosclerosis, rigorous risk stratification for CAD is necessary in subjects with established diabetes irrespective of the obesity status in the Korean population.

Although obesity is associated with an increased risk of cardiovascular disease [32, 33], several studies have suggested an interesting phenomenon, the obesity paradox, which is the protective effect of obesity against adverse clinical outcomes in patients with obstructive CAD [34–36]. However, these studies evaluated the impact of obesity on prognosis only in patients with established CAD. In the present study, diabetes was independently associated with the presence and severity of CAD, but obesity was not independently associated with CAD in subjects with established diabetes. Although the relationship between obesity and prognosis in subjects with diabetes remains uncertain in the Asian population, it may be more important to predict the development of CAD in non-obese subjects with diabetes compared with their obese counterparts considering the protective effect of obesity in subjects with obstructive CAD. Further prospective studies with larger sample sizes are necessary to address this issue.

### Limitations

Several limitations should be acknowledged in the present study. First, we could not eliminate the possible effects of underlying medication for hypertension, dyslipidemia, and diabetes on coronary atherosclerosis because of the observational design of this study. Second, we used only the criterion of BMI ≥25 kg/m^2^ for defining the obesity status. Although it is well-known that BMI is significantly associated with abdominal fat and waist circumference in the Korean population [37], further evaluation of the association between other anthropometric indices and coronary atherosclerosis according to the presence of diabetes may be necessary in Asian populations.

Despite these limitations, it is novel in that only participants of Asian ethnicity were evaluated in the present study compared with other studies performed in Western populations. In addition, the clinical usefulness of obesity for predicting CAD according to the diabetes status was first evaluated in approximately 7,300 Asian subjects with near-normal kidney function using large-scale CT data. The findings of this study may be helpful to identify the differential association between obesity and CAD according to the diabetes status in the Asian population.

## Conclusions

Despite a significantly higher prevalence of obesity in diabetic subjects than in non-diabetic subjects, the majority of the diabetic subjects were non-obese in our Korean population. Obesity has an independent predictive value for the presence of CAD and severe coronary calcification only in subjects without established diabetes. Considering the incremental impact of diabetes, rigorous risk stratification for CAD might be necessary in diabetic subjects irrespective of the obesity status among Asian population.

## Abbreviations

BMI: Body mass index
CACS: Coronary artery calcium score
CAD: Coronary artery disease
CCTA: Coronary computed tomographic angiography
CAD: Coronary artery disease
CI: Confidence intervals
CMP: Calcified or mixed plaque
GFR: Glomerular filtration rate
HDL: High-density lipoprotein
LDL: Low-density lipoprotein
MDCT: Multidetector computed tomography
NCP: Non-calcified plaque
OR: Odds ratios.
